# Author Correction: Development of a model-inference system for estimating epidemiological characteristics of SARS-CoV-2 variants of concern

**DOI:** 10.1038/s41467-021-27703-9

**Published:** 2021-12-16

**Authors:** Wan Yang, Jeffrey Shaman

**Affiliations:** 1 Department of Epidemiology, Mailman School of Public Health, Columbia University, New York, NY USA; 2 Department of Environmental Health Sciences, Mailman School of Public Health, Columbia University, New York, NY USA

**Keywords:** Computational biology and bioinformatics, SARS-CoV-2, Infectious diseases, Epidemiology

Correction to: *Nature Communications* 10.1038/s41467-021-25913-9, published online 22 September 2021.

The original version of this article contained errors in the Supplementary Information, equations S3 and S5.

Equation S3, line 4, was missing a term. The original read:

$$\left\{\begin{array}{c}\frac{{dS}}{{dt}}=\frac{R}{L}-\frac{{{b}_{t}{e}_{t}{m}_{t}\beta }_{t}{IS}}{N}-\varepsilon -{v}_{1}(t)-{v}_{2}(t)\\ \frac{{dE}}{{dt}}=\frac{{{b}_{t}{e}_{t}{m}_{t}\beta }_{t}{IS}}{N}-\frac{E}{Z}+\varepsilon \hfill\\ \,\frac{{dI}}{{dt}}=\frac{E}{Z}-\frac{I}{D}\hfill\\ \frac{{dR}}{{dt}}=\frac{I}{D}+{v}_{1}\left(t\right)+{v}_{2}(t)\hfill\end{array}\right.$$

This has been corrected to:

$$\left\{\begin{array}{c}\frac{{dS}}{{dt}}=\frac{R}{L}-\frac{{{b}_{t}{e}_{t}{m}_{t}\beta }_{t}{IS}}{N}-\varepsilon -{v}_{1}(t)-{v}_{2}(t)\\ \frac{{dE}}{{dt}}=\frac{{{b}_{t}{e}_{t}{m}_{t}\beta }_{t}{IS}}{N}-\frac{E}{Z}+\varepsilon \hfill\\ \,\frac{{dI}}{{dt}}=\frac{E}{Z}-\frac{I}{D}\hfill\\ \frac{{dR}}{{dt}}=\frac{I}{D}-\frac{R}{L}+{v}_{1}\left(t\right)+{v}_{2}(t)\hfill\end{array}\right.$$

Equation S5, line 2 was incorrectly written; the original read:

$$\left\{\begin{array}{c}\frac{{{dS}}_{i}^{A}}{{dt}}=\frac{{R}_{i}^{A}}{{L}_{i}^{A}}-\mathop{\sum}\limits_{j}{{b}_{t}{e}_{t}{m}_{t}c}_{{ij}}\mathop{\sum}\limits_{a}\frac{{\beta }_{j}^{{Aa}}{S}_{j}^{A}{I}_{j}^{a}}{{N}^{a}}-{\varepsilon }_{i}-{v}_{i,1}^{A}(t)-{v}_{i,2}^{A}(t)\\ \frac{{{dE}}_{i}^{A}}{{dt}}=\mathop{\sum}\limits_{j}{b}_{t}{e}_{t}{m}_{t}\mathop{\sum}\limits_{a}\frac{{\beta }_{j}^{{Aa}}{S}_{j}^{A}{I}_{j}^{a}}{{N}^{a}}-\frac{{E}_{i}^{A}}{{Z}_{i}^{A}}+{\varepsilon }_{i}\hfill\\ \frac{{{dI}}_{i}^{A}}{{dt}}=\frac{{E}_{i}^{A}}{{Z}_{i}^{A}}-\frac{{I}_{i}^{A}}{{D}_{i}^{A}}\hfill\\ \frac{{{dR}}_{i}^{A}}{{dt}}=\frac{{I}_{i}^{A}}{{D}_{i}^{A}}-\frac{{R}_{i}^{A}}{{L}_{i}^{A}}+{v}_{i,1}^{A}\left(t\right)+{v}_{i,2}^{A}(t)\hfill\end{array}\right.$$

This has been corrected to:

$$\left\{\begin{array}{c}\frac{{{dS}}_{i}^{A}}{{dt}}=\frac{{R}_{i}^{A}}{{L}_{i}^{A}}-\mathop{\sum}\limits_{j}{{b}_{t}{e}_{t}{m}_{t}c}_{{ij}}\mathop{\sum}\limits_{a}\frac{{\beta }_{j}^{{Aa}}{S}_{j}^{A}{I}_{j}^{a}}{{N}^{a}}-{\varepsilon }_{i}-{v}_{i,1}^{A}(t)-{v}_{i,2}^{A}(t)\\ \frac{{{dE}}_{i}^{A}}{{dt}}={b}_{t}{e}_{t}{m}_{t}\mathop{\sum}\limits_{a}\frac{{\beta }_{i}^{{Aa}}{S}_{i}^{A}{I}_{i}^{a}}{{N}^{a}}-\frac{{E}_{i}^{A}}{{Z}_{i}^{A}}+{\varepsilon }_{i}\hfill\\ \frac{{{dI}}_{i}^{A}}{{dt}}=\frac{{E}_{i}^{A}}{{Z}_{i}^{A}}-\frac{{I}_{i}^{A}}{{D}_{i}^{A}}\hfill\\ \frac{{{dR}}_{i}^{A}}{{dt}}=\frac{{I}_{i}^{A}}{{D}_{i}^{A}}-\frac{{R}_{i}^{A}}{{L}_{i}^{A}}+{v}_{i,1}^{A}\left(t\right)+{v}_{i,2}^{A}(t)\hfill\end{array}\right.$$

These corrections have been made to the Supplementary Information pdf.

## Supplementary information

Updated Supplementary Information

