# Supplementary material for: Development of a model-inference system for estimating epidemiological characteristics of SARS-CoV-2 variants of concern
Source: Nat Commun. 2021 Sep 22;12:5573. doi: 10.1038/s41467-021-25913-9 (PMC8458278; doi:10.1038/s41467-021-25913-9)
Supplement: Supplementary file 1 — Supplementary Information [file 41467_2021_25913_MOESM1_ESM.pdf]

Development of a model-inference system for estimating epidemiological characteristics of SARS-CoV-2 variants of concern

Wan Yang[1] and Jeffrey Shaman[2]
[1]Department of Epidemiology, [2]Department of Environmental Health Sciences, Mailman School of Public Health, Columbia University, New York, NY, USA

Supplementary Methods
Supplementary Figures 1 – 7
Supplementary Tables 1 – 5

Supplementary Methods

1. Estimating seasonal trends

Many respiratory infections tend to occur seasonally and are predominantly prevalent during certain months of the year (e.g., cold months in temperate climates).[1] This seasonal pattern has been documented for influenza viruses,[2] respiratory syncytial viruses,[3] and endemic human coronaviruses.[4] In addition, studies have showed that this seasonality may be associated with climate conditions – particularly, temperature and humidity – as they may modulate the survival and transmission of respiratory viruses.[5, 6, 7, 8] For the SARS-CoV-2 virus, our work has also shown that a winter-time seasonality exists, similar to endemic human coronaviruses in New York City (NYC), and that models accounting for this seasonality enable more accurate projection of COVID-19 pandemic dynamics than those do not.[9, 10] However, to date, no mechanistic models exist that quantify the response of the SARS-CoV-2 virus to temperature and humidity and in turn the seasonality of COVID-19. In addition, seasonal trends may differ by climate. For instance, epidemics of influenza can occur any time of the year in subtropical and tropical climates; it is thus more difficult to characterize the seasonality of respiratory infections in these climates. To address these challenges, we recently developed a flexible climate-forced model of epidemic dynamics for subtropical and tropical climates; results with this model also describe the response to temperature and humidity conditions common in temperate climates.[11] Thus, to account for the potentially diverse seasonal trends of COVID-19 in the UK (temperate climates), South Africa (mostly temperate climates), and Brazil (mostly tropical climates), we applied this climate-forcing to temperature and humidity data for each country and computed the relative seasonal trend for each country.

Specifically, the climate-forcing takes the following form:

$$R_0(t) = [aq^2(t) + bq(t) + c][\frac{T_c}{T(t)}]^{T_{exp}} \qquad \text{[S1]}$$

where $R_0(t)$ is the basic reproduction number at time t; $q(t)$ is specific humidity (i.e. a measure of absolute humidity) at time t; and $T(t)$ is temperature at time t. In essence, the forcing function assumes that specific humidity has a bimodal effect on R_0, with both low and high humidity conditions favoring transmission; in addition, this effect is moderated by temperature, where low temperatures promote transmission and temperatures above a certain threshold (i.e., T_c in Eqn. S1) limit transmission. Further, to link the coefficients a, b, and c to humidity q and R_0, Yuan et al.[11] reparametrized the forcing function by solving the parabola with a nadir at (q_{mid}, R_{0max} -R_{0diff}) and maxima at both (q_{min}, R_{0max}) and (q_{max}, R_{0max}), such that:

$$\begin{cases} a = \dfrac{-b}{q_{max} + q_{min}} \\ b = \dfrac{R_{0_{diff}}(q_{max} + q_{min})}{(q_{max} - q_{mid})(q_{min} - q_{mid})} \\ c = \left(R_{0_{max}} - R_{0_{diff}}\right) - aq_{mid}^2 - bq_{mid} \end{cases} \quad [S2]$$

Yuan et al.[11] estimated the parameters R_{0max} (i.e., the maximum R_0), R_{0diff} (i.e., the difference between the maximum and minimum R_0), q_{min}, q_{mid}, and q_{max} (i.e., the minimum, median, and maximum specific humidity for the response), T_c (the threshold temperature) and T_{exp} (the exponent in Eqn S1) for influenza in Hong Kong, a subtropical city, based on long-term epidemic data collected therein during 1998 - 2018. Here we use their mean estimates for these parameters and temperature and humidity data for each country (see main text and Supplementary Fig 7) to compute the seasonal trend for each country using Eqns. S1-2. However, as these parameters were estimated for influenza, the outputs do not represent the actual R_0 for the SARS-CoV-2 virus. Thus, we instead compute the *relative* seasonal trend, by scaling the weekly country output from Eqn S1 by the country mean output, such that this scaled output provides a *relative*, seasonality-related transmissibility for each week of the year. These relative estimates also decouple the seasonality-related and variant-specific transmissibility (assuming no interaction; see below). The estimated seasonal trends are shown in Supplementary Fig 2 and used in the epidemic model to represent disease seasonality for each country via the parameter b_t in Eqn S3 (see below).

2. Model-inference system

The model-inference system developed for this study consists of an SEIRSV model to simulate the transmission dynamics of SARS-CoV-2 and the ensemble Kalman adjustment filter (EAKF)[12] to estimate the model state variables and parameters, based on case and mortality data. Here we describe the model and the filtering method in detail.

2.1. Epidemic model.

The SEIRSV (susceptible-exposed-infectious-removed-susceptible-vaccination) model uses the following set of equations to simulate the transition of sub-populations between different disease stages, while accounting for disease seasonality, concurrent non-pharmaceutical interventions (NPIs), and vaccination:

$$
\begin{cases}
\frac{dS}{dt} = \frac{R}{L} - \frac{b_t e_t m_t \beta_t IS}{N} - \varepsilon - v_1(t) - v_2(t) \\
\frac{dE}{dt} = \frac{b_t e_t m_t \beta_t IS}{N} - \frac{E}{Z} + \varepsilon \\
\frac{dI}{dt} = \frac{E}{Z} - \frac{I}{D} \\
\frac{dR}{dt} = \frac{I}{D} - \frac{R}{L} + v_1(t) + v_2(t)
\end{cases} \qquad \text{[S3]}
$$

where S, E, I, R are the number of susceptible, exposed (but not yet infectious), infectious, and removed (i.e., recovered or immune) individuals, respectively; N is the population size. The parameter ε represents travel-related importation of infections (nominally set to 1 per 20 days per 1 million population, unless specified otherwise).

To account for local seasonality, b_t, the estimated relative seasonal trend for each country (see Section 1 above and Supplementary Fig 2), is used to adjust the relative transmission rate at time t. For instance, for the UK (Supplementary Fig 2A), the estimated seasonal trend b_t is 1.15 for week 1 (a week during winter), indicating the transmission rate for that week is 1.15 times the yearly average, due to the more favorable winter weather conditions; similarly, estimated b_t is 0.86 for week 27 (a week during summer), indicating the transmission rate for that week is 86% of the yearly average, due to the less favorable summer weather conditions.

Different NPIs (e.g., stay-at-home mandate for nonessential workers, school closures, masking mandate, and other social distancing rules and preventive measures) have been implemented during the COVID-19 pandemic. However, the specific NPIs implemented varied by location and time; in addition, the effectiveness of each NPI remains unclear. It is thus difficult to detail and capture specific NPI and effectiveness in the model. Nevertheless, previous studies have shown that the overall impact of NPIs (e.g., reduction in the reproduction number R_t) has been highly correlated with population mobility during the COVID-19 pandemic.[9, 13 14] As such, here we used the relative mobility as observed in each study location to represent the overall impact of NPIs. Specifically, to account for concurrent NPIs, the term m_t, the relative population mobility at time t (in this study, we use data from Google Community Mobility Reports;[15] see main text and Supplementary Fig 2), is used to adjust the transmission rate. For instance, for the UK (Supplementary Fig 2A), relative mobility m_t was 41% of pre-COVID levels for the week starting March 22, 2020; per Eqn 3, the overall transmission rate for that week was reduced by 41%, *before* adjusting for the effectiveness. To further account for the potential changes in effectiveness, the model additionally includes a parameter, e_t, to scale NPI effectiveness at time t. This effectiveness (e_t) was then estimated by the model-inference system along with other model variables and parameters.

For virus-specific characterization, β_t is the variant-specific transmission rate at time t, Z is the latency period, D is the infectious period, and L is the immunity period. Note that the parameters e_t, β_t, Z, D, and L are estimated by the model-inference system as described below.

To incorporate changes in population susceptibility due to vaccination, the model accounts for two-dose vaccination via $v_1(t)$ and $v_2(t)$. Specifically, $v_1(t)$ is the number of individuals *successfully* immunized after the first dose of the vaccine and is computed using vaccination data and vaccine efficacy for one dose (see detailed settings in Supplementary Table 2). Similarly, $v_2(t)$ is the additional number of individuals successfully immunized after the second vaccine dose (excluding those successfully immunized after the first dose). As an example, assume 100 individuals receive their 1st dose on day 0 and 2nd dose on day 21 and the vaccine efficacy (VE) is 85% 14 days after the 1st dose and 95% 7 days after the 2nd dose. To compute v_1 and v_2, we first adjust for immunity gained from infection; here for illustration, assume 10% have immunity from prior infection at time of vaccination (for illustration, assumed the same for both doses here; in this study, these were based on model-estimated cumulative infection rate and adjusted for waning immunity), then the number of individuals susceptible at time of vaccination would be 100 × (1 – 10%) = 90. Further adjusting for the time lag of immunity development and VE, $v_1(t = \text{day } 14)$ is calculated as 90 × 85% = 76.5 and v_2 (t = day 28) is calculated as 90 × (1 – 85%)[1 – (1 – 85%)(1 – 95%)] = 9, such that the total percentage of individuals successfully immunized is (76.5 + 9) / 90 = 95%, i.e. VE for the fully vaccinated.

2.2. Observation model: accounting for under-detection of infections and delay in detection. We compute the model-simulated number of cases and deaths for each week using the model-simulated infection rate, as done in Yang et al.[10] Specifically, we include 1) a time-lag from infectiousness to detection (i.e., an infection being diagnosed as a case) – drawn from a gamma distribution with a mean of T_m and standard deviation (SD) of T_{sd} days – to account for delays in diagnosis and detection; 2) an infection-detection rate (r), i.e. the fraction of infections (including subclinical or asymptomatic infections) reported as cases, to account for under-detection; 3) a time-lag from infectiousness to death, drawn from a gamma distribution with a mean of 14 days and a standard deviation of 10 days, empirically based on mortality data; and 4) an infection-fatality risk (IFR), i.e., the fraction of infections that die from COVID-19. To compute the model-simulated number of new cases per week, we multiply the model-simulated number of new infections per day by the infection-detection rate, and further distribute these simulated cases in time per the distribution of time-from-infectiousness-to-detection. We then aggregate the daily lagged, simulated cases to weekly totals for model inference (see below). Similarly, to compute the model-simulated deaths per week and account for delays in time to death, we multiply the simulated-infections by the IFR and then distribute

these simulated deaths in time per the distribution of time-from-infectiousness-to-death, and aggregate these daily numbers to weekly totals. For each week, the infection detection rate (r), the mean (T_m) and standard deviation (T_{sd}) of time-from-infectiousness-to-detection, and the IFR are estimated based on weekly case and mortality data, along with other model parameters.

2.3. Inference using the EAKF

At the end of each week, the inference system uses the EAKF to update the state variables and parameters based on model-generated prior estimates and case and mortality data. Briefly, the EAKF uses an ensemble of model realizations (n=500 here), each with initial parameters and variables randomly drawn from a prior range (see Supplementary Table 2). After model initialization, the system integrates the model ensemble forward in time for a week (per Eqn S3) *stochastically* to compute the prior distribution for each model state variable or parameter, as well as the model-simulated number of cases and deaths for that week as described in Section 2.2. The system then combines the prior estimates with the observed case and death data for the same week to compute the posterior per Bayes' theorem.[12] During this filtering process, the system updates the posterior distribution of all model parameters and variables for each week.[12] As such, it is able to capture the time-varying changes in transmission dynamics including the variant-specific transmission rate (β_t) and infectious period (D) – the two parameters that we use to compute variant-specific transmissibility over time.

However, unlike previous studies using similar model-inference approaches, here we further modify the EAKF filtering process to test different potential combinations of changes in transmissibility and immune escape. To enable this exploration of systemic changes (e.g. due to the emergence of a new variant), we randomly replace a small fraction of ensemble members (3-10%) using values randomly drawn from specified ranges. This technique, termed space reprobing (SR), was developed in order to explore state space without corrupting performance of the filter.[16] Specifically for this application, we apply SR to a given related set of parameters/variables and restrict the EAKF update of non-related parameters/variables, for 14 different hypothesized behaviors. These hypothesized changes are as follows:

1) Hypothesis 1 (minor changes in transmissibility, no immune escape): Large updates are only allowed for the two transmissibility-related parameters β_t and D; to explore the changes, the system applies SR to these two parameters using values drawn from prior ranges 10-20% higher than the initial priors.
2) Hypothesis 2 (major changes in transmissibility only, no immune escape): Similar to 1); but to explore the changes, the system applies SR to β_t and D using values drawn from prior ranges 30-40% higher than the initial priors.

3) Hypothesis 3 (minor immune escape only, no changes to transmissibility): Large updates are only allowed for *S*, the population susceptibility, up to a total loss of 50% of the prior immunity.
4) Hypothesis 4 (major immune escape only, no changes to transmissibility): Large updates are only allowed for *S*, the population susceptibility, up to a total loss of 95% of the prior immunity.
5) Hypothesis 5 (minor changes in transmissibility + minor immune escape): combining 1) and 3) above.
6) Hypothesis 6 (major changes in transmissibility + major immune escape): combining 2) and 4) above.
7) Hypothesis 7 (minor changes in transmissibility + major immune escape): combining 1) and 4) above.
8) Hypothesis 8 (major changes in transmissibility + minor immune escape): combining 2) and 3) above.
9) Hypothesis 9 (changes in both transmissibility and immune escape, no restriction on magnitude of change): Large updates are allowed for β_t and *D* as well as *S*. To explore the changes, initial SR uses values drawn from prior ranges 10-20% higher than the initial priors, and values up to 30-40% higher than the initial priors if the inference system detects the prior continues to underestimate the observed cases and deaths with the 10-20% initial increase in SR values. In addition, SR allows updates of *S* up to 95% of the prior immunity.

To account for slower changes in overall population immunity (i.e., in the entire country) as the new variant gradually spreads to different sub-regions across a large geographic space, such as in Brazil, we also explore the fitting using the following five additional settings:

10) Hypothesis 10 (immune escape only and changes to overall population immunity occur slowly over time): Large updates are only allowed for *S*, up to a total loss of 95% of the prior immunity; however, SR is applied to a smaller fraction of ensemble members than in 1)-9) such that changes in *S* occur gradually.
11) Hypothesis 11 (minor changes in transmissibility + minor immune escape; both occur slowly over time): Large updates are allowed for β_t and *D* as well as *S*. Adjustment to *S* is allowed as in 10) but up to only 50% of prior immunity. In addition, for transmissibility, the system applies SR to β_t and *D* using values drawn from prior ranges 10-20% higher than the initial priors.
12) Hypothesis 12 (major changes in transmissibility + minor immune escape; both occur slowly over time): Similar to 11); however, for β_t and *D*, initial SR uses values drawn from prior ranges 10-20% higher than the initial priors, and values up to 30-40% higher

than the initial priors if the inference system detects the prior continues to underestimate the observed cases and deaths with the 10-20% initial increase in SR values.

13) Hypothesis 13 (minor changes in transmissibility + major immune escape; both occur slowly over time): Similar to the settings specified in 11) but adjustment to *S* is allowed up to 95% of prior immunity.
14) Hypothesis 14 (major changes in transmissibility + major immune escape; both occur slowly over time): Similar to settings specified in 12) but adjustment to *S* is allowed up to 95% of the prior immunity.

We carry out the model-inference process for each of the 14 settings described above and for each country dataset. We then select the most plausible hypothesis for each country based on the following criteria: 1) model fitting to case and mortality data, as indicated by the relative root-mean-squared-error (RRMSE) between the *posterior* estimates for the corresponding variable (i.e. case rate or mortality rate) and data; 2) the accuracy of one-step ahead prediction, as indicated by the RRMSE between the *prior* estimates for the corresponding variable (i.e. case rate or mortality rate) and data; 3) the level of adjustment needed for two key variables, i.e., infection rate and case rate, as indicated by the RRMSE between the *prior* and *posterior* estimates for each variable; 4) a penalty on the number of variables needing SR adjustment; and 5) a penalty on the frequency of SR adjustment. We combine all these metrics by weighting them heuristically using the following set of weights: 0.27 for the two metrics in 1); 0.13 for the two metrics in 2), 0.03 for the two metrics in 3), and 0.07 for both 4) and 5). We also tested other sets of weights and found that higher weights should be given to 1) and 2) based on results from the synthetic testing where the ‘true’ values of the state variables and parameters are known; however, in general, the final results are similar if there are minor changes to these weights.

To account for model stochasticity, we repeat each model-inference 100 times for each dataset, each with initial parameters and variables randomly drawn from the prior distributions (Supplementary Table 2). Each model-inference tests the 14 hypotheses described above, selects the one with the best performance (i.e. minimizing the combined metric described above), and outputs the estimates of the best-performing run. That is, the model estimates reported in the main text are aggregated from 100 best-performing model runs (each with 500 ensemble members and totaling 50,000 individual model realizations).

3. Model validation using model-generated synthetic data

To test the accuracy of our model-inference system, we generate 10 synthetic datasets using a separate multi-variant SEIRS model, similar to models developed in Yang et al.[17] and Gog &

Grenfell.[18] Within this model, variants can interact via cross-immunity, which protects a portion of individuals with prior infection (i.e. polarized immunity) by reducing transmission. Specifically, the model takes the following form:

$$\begin{cases} \frac{dS_i}{dt} = \frac{R_i}{L_i} - \sum_j \frac{b_t e_t m_t c_{ij} \beta_j S_j I_j}{N} - \varepsilon \\ \frac{dE_i}{dt} = \frac{b_t e_t m_t \beta_i S_i I_i}{N} - \frac{E_i}{Z_i} + \varepsilon \\ \frac{dI_i}{dt} = \frac{E_i}{Z_i} - \frac{I_i}{D_i} \\ \frac{dR_i}{dt} = \frac{I_i}{D_i} - \frac{R_i}{L_i} \end{cases} \quad \text{[S4]}$$

where N is the population size; S_i, E_i, I_i, and R_i, are, respectively, the numbers of susceptible, exposed-but-not-yet infectious, infectious, and recovered individuals, with respect to variant-*i* (here, the wild-type SARS-CoV-2 virus or a new variant); β_i, D_i, and L_i are, respectively, the transmission rate, mean infectious period, and mean immunity period, for variant-*i*; and c_{ij} measures the strength of cross-immunity to variant-*i* conferred by infection with variant-*j* (e.g., close to 0 if it is weak and c_{ii}=1 for infection by the same variant). The parameter ε represents travel-related importation of infections; to generate the synthetic data (i.e., "truths"), we set ε to 1 per week for the first 5 weeks and 1 every 3 days for the rest of the first wave (here weeks 1-20); for the second wave, as transmission has been established locally, we set ε to 0 for simplicity. The terms b_t, m_t, and e_t are the same as in Eqn S3 and account for seasonality and NPIs over time. For simplicity, we omit birth, death, and vaccination.

To generate the synthetic data (i.e., "truths"), we seed the Eq. S4 model with 2 infections of wild-type virus at the start of each simulation and 50 infections of a new variant at the start of Week 21, for N = 1 million people; we run the model stochastically with a daily time-step from the week starting 3/1/2020 to the week starting 2/21/2021 (i.e. 52 weeks in total) using the parameters listed in Supplementary Table 3. To compute the weekly number of cases and deaths, we use the same procedure as described in Section 2.2 above for each variant. We then combine the case/mortality estimates for both variants, add random noises drawn from a Poisson distribution to mimic observational error. The final noise-added weekly case and mortality time series are then used as synthetic data for testing the model-inference system (described in Section 2 above). To compare the posterior estimates of key parameters and variables (e.g. transmissibility and population susceptibility) from the model-inference system, we compute the true values of population susceptibility and transmissibility over time as the weighted average of the two variants based on the relative prevalence during each week. Fig 1

and Supplementary Fig 1 show the 10 model-generated truths including cases, deaths, and the computed "true" values of population susceptibility and transmissibility for each week of the simulation.

4. Multi-variant, age-structured model for simulation to test the relative competitiveness of VOCs and project future SARS-CoV-2 dynamics.

We modify the multi-variant model in Eqn S4 to further include age structure and vaccination. The inclusion of age structure here allows incorporation of age-specific parameters (e.g., transmission rate and infection-fatality risk) as well as age-specific vaccination coverage and rates. Specifically, this multi-variant, age-structured SEIRSV model takes the following form:

$$\begin{cases} \frac{dS_i^A}{dt} = \frac{R_i^A}{L_i^A} - \sum_j b_t e_t m_t c_{ij} \sum_a \frac{\beta_j^{Aa} S_j^A I_j^a}{N^a} - \varepsilon_i - v_{i,1}^A(t) - v_{i,2}^A(t) \\ \frac{dE_i^A}{dt} = b_t e_t m_t \sum_a \frac{\beta_i^{Aa} S_i^A I_i^a}{N^a} - \frac{E_i^A}{Z_i^A} + \varepsilon_i \\ \frac{dI_i^A}{dt} = \frac{E_i^A}{Z_i^A} - \frac{I_i^A}{D_i^A} \\ \frac{dR_i^A}{dt} = \frac{I_i^A}{D_i^A} - \frac{R_i^A}{L_i^A} + v_{i,1}^A(t) + v_{i,2}^A(t) \end{cases} \qquad [S5]$$

Model parameters in Eqn S5 are similar to those in Eqn S4, except for those related to age, which are indicated by the superscripts. The formulation of the age-related model component follows a typical age-structured model; for a given age group *A* (designated by the superscript), the total infection rate by a given variant-*i* (designated by the subscript) is the sum over all age groups (i.e., $\sum_a \frac{\beta_i^{Aa} S_i^A I_i^a}{N^a}$, where β_i^{Aa} is the transmission rate of variant-*i* from age-group *a* to age-group *A*). The vaccination model component is similar to Eqn S3, but with age-stratification. However, the terms $v_{i,1}^A(t)$ and $v_{i,2}^A(t)$ are variant-specific, as indicated by the additional subscript *i*; that is, they additionally account for the reduction in vaccine efficacy against the new variants, based on scenario assumptions specified in Supplementary Table 5.

As an example, we simulate the transmission dynamics under different scenarios of variant prevalence, vaccine efficacy, and NPIs for a city like NYC, from the week of 4/25/2021 to the week of 8/22/2021 (i.e., approximately May - August 2021). We use data or estimates available for NYC to initialize the parameters and state variables needed for model simulations. In addition, we use our model-inference estimates for the VOCs for related parameters and variables. Specifically, as in previous work,[9, 10, 19] we include 8 age-groups (i.e. <1, 1-4, 5-14, 15-24, 25-44, 45-64, 65-74, and 75+ year-olds) to account for age-specific differences. To focus on the three VOCs, here we only include the B.1.1.7, B.1.351, and P.1 variants and attribute the

rest as “wildtype” virus. NYC data on variant prevalence among tested infections during the weeks of March – April 2021 are used to inform the initial range of seeding for each variant (Supplementary Table 5). Initial conditions for the state variables (e.g., susceptibility and SARS-CoV-2 prevalence) for each age group are taken from estimates[9, 10, 19] made for the week of 4/18/2021, using detailed data (including case, mortality, COVID-19-related emergency visit, mobility, and vaccination) during 3/1/2020 – 4/24/2021. For each age group, to compute the initial variant-specific population susceptibility (S_i) at the start of a simulation, we move the estimated proportion with immune escape for variant *i* among those who have had prior infection with the wild-type virus but have not been vaccinated back to the susceptible compartment. The number of people losing vaccine-induced immunity is computed based on scenario assumptions determining the reduction in vaccine efficacy (see scenarios in Supplementary Table 5).

The cross-immunity settings, i.e., values of c_{ij}'s in Eqn S5, come from our posterior model-inference estimates of immune escape and are used for all age groups. To reduce uncertainty, here we use the 80% CI estimates (see Supplementary Table 5). For instance, as our model-inference estimate of immune escape for B.1.351 has an 80% CI of 40.1 – 82.8%, we set *c*(B.1.351←wildtype), the cross-immune protection against B.1.351 conferred by prior infection of the wildtype virus relative to variant-specific immunity, to values drawn from a uniform distribution ranging from 0.172 to 0.599 (i.e. cross-immunity is set to the complement of estimated immune escape). We set all *c*(wildtype←new variant) to 1 – that is, we assume full cross-immune protection against the wild-type virus conferred by infection due to any VOC.

Similarly, the variant-specific transmission rates, i.e. β_i's, come from our posterior model-inference estimates of the relative transmissibility for each variant. For instance, as our model-inference estimate of transmissibility for B.1.351 is 18.5 – 45.7% (80% CI) higher than that of the wildtype virus, we set $\beta_{\text{B.1.351}}$ to 1.185 – 1.457 times of the estimate for β_{wildtype}. The same scaling is applied to all age groups.

Due to a lack of information, we do not account for potential differences in infection fatality risk by variant; therefore, the simulated mortality under different scenarios only reflect the relative infection rate by age group, for which we apply age-specific infection-fatality risk (see Supplementary Table 5). In addition, due to the uncertainty of the infection fatality risk among breakthrough infections (i.e., those who have been vaccinated), we only show mortality-related simulations for the “Same VE” scenario which assumes no reduction in VE.

We run the model for each scenario 1000 times stochastically, with the parameters and initial conditions randomly drawn from uniform distributions with ranges specified in Supplementary Table 5. Results are summarized from the 1000 model runs for each scenario.

Supplementary Fig 1. Model-inference system validation using model-generated synthetic data with an infection-detection rate of 10%. For this testing, the true values of incidence and mortality by week (A), transmissibility by week (B, top panel), population susceptibility by week (b, bottom), and overall changes in transmissibility and immune escape due to a new variant (C) were generated by model simulations with prescribed parameters and conditions. Unlike the real-world in which most epidemiological characteristics are unobserved, here these quantities (i.e. the 'Truth') are prescribed and known and thus can be compared to estimates made with the model-inference system using the synthetic, model-generated incidence and mortality data (A). (A) 5 sets of synthetic data (dots) and model-fits to each dataset; lines show mean estimates and surrounding areas show 50% (dark) and 95% (light) CrIs. (B) Weekly model estimated transmissibility and population susceptibility. The lines show mean estimates and surrounding areas show 50% (dark) and 95% (light) CrIs, compared to the true values (dots). (C) overall estimates of the change in transmissibility ('Trans') and immune escape ('Imm esc'), compared to the true values (dots); boxes show model estimated median (middle bar) and interquartile range (box edges) and whiskers show model estimated 95% CIs, from n=100 model-inference simulations.

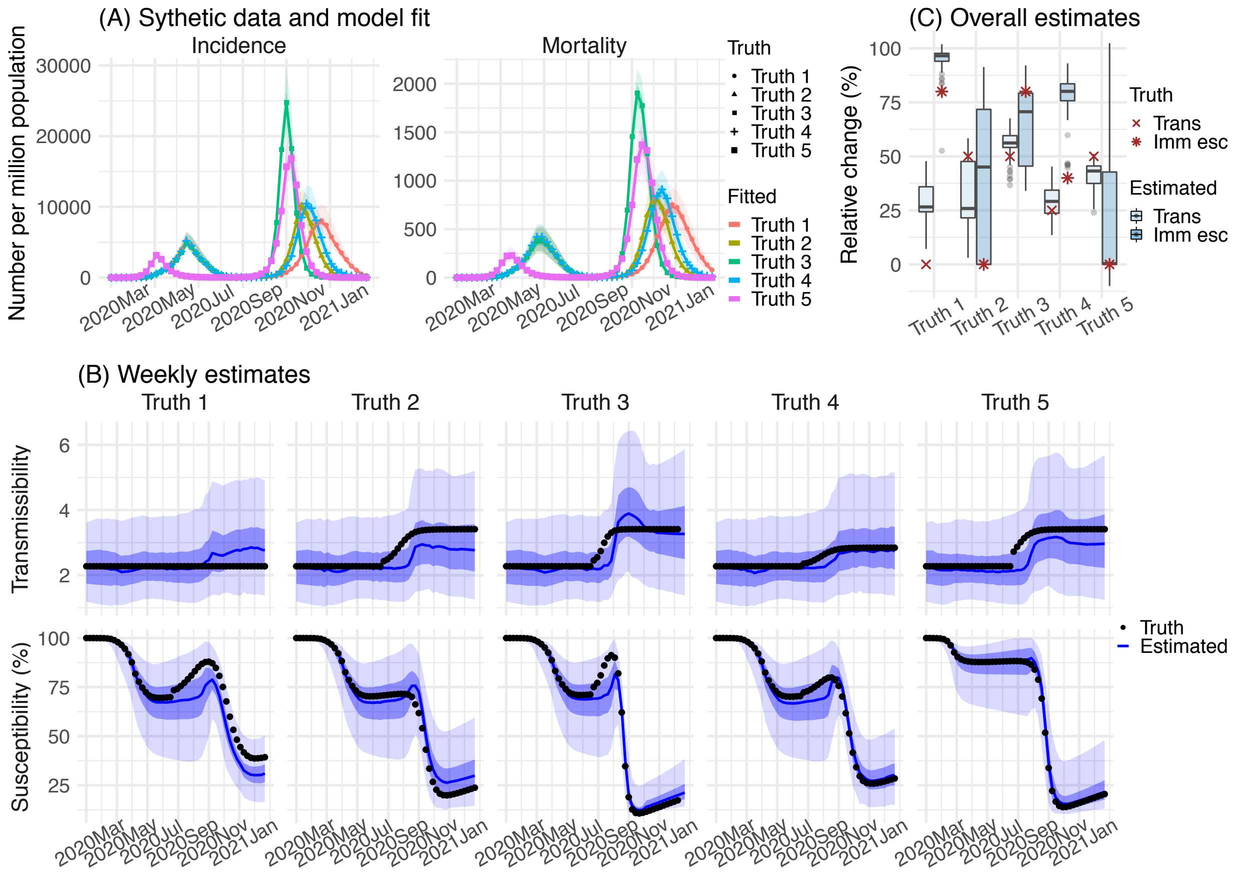

Supplementary Fig 2. Pandemic dynamics, mobility, and estimated seasonal trends, and vaccination uptake in the three countries: (A) United Kingdom, (B) South Africa, and (C) Brazil. Bars show weekly numbers of reported cases per 1 million persons (grey) and reported deaths per 100,000 persons (red). Green lines show relative mobility by week (relative to pre-pandemic levels), based on data from Google Community Mobility Reports (see main text, Methods, “Data sources and processing”). Orange lines show estimated seasonal trends (see SI, Section 1, “Estimating seasonal trends”). Blue lines show the cumulative fractions of population partially vaccinated (dashed lines) and fully vaccinated (solid lines), based on vaccination data from each country (see main text, Methods, “Data sources and processing”).

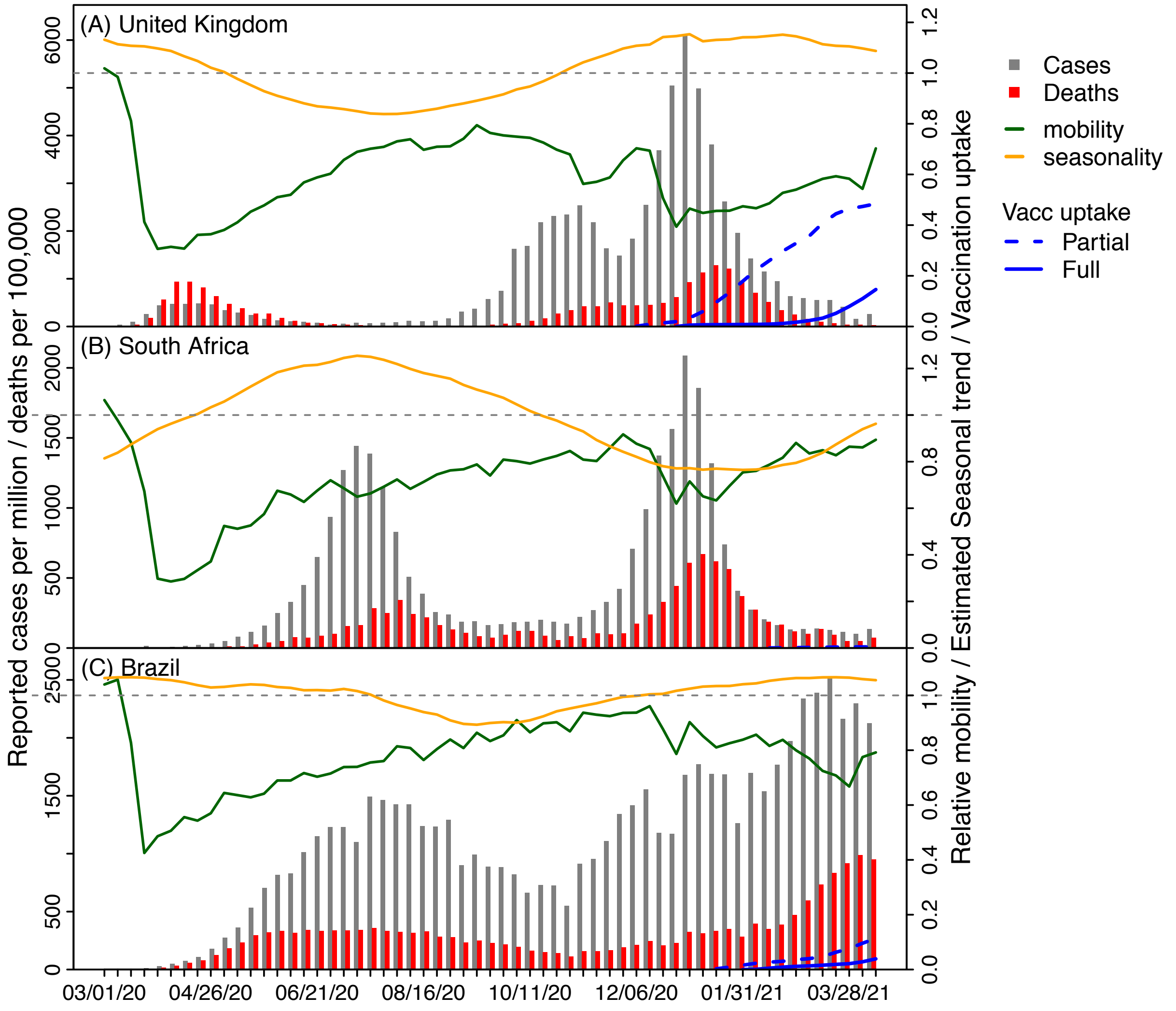

Supplementary Fig 3. Other model estimates. Left panel (A, C, and E) shows the estimated infection-detection rate and right panel (B, D, and F) shows the estimated infection-fatality risk for each week during the study period for the three countries. For comparison, estimated weekly infection rates are superimposed in each plot (right y-axis). Blue lines and surrounding areas show model estimated mean, 50% and 95% CrIs. Boxes (middle bar = mean; edges = 50% CrIs) and whiskers (95% CrIs) show the estimated weekly infection rates. Grey shaded boxes indicate the timing of lockdowns or key period of restrictions; horizontal arrows indicate the timing of variant identification and vaccination rollout.

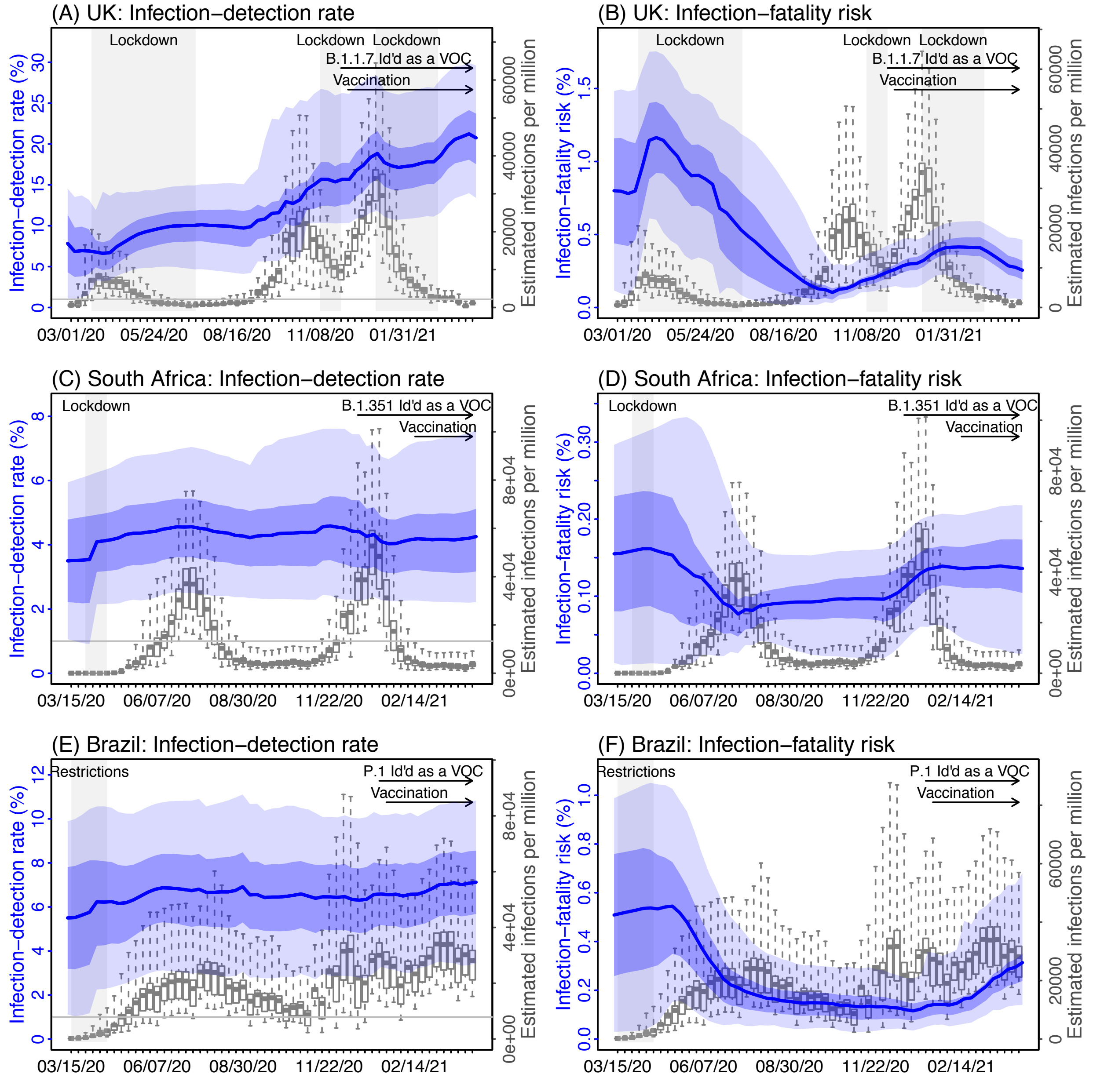

Supplementary Fig 4. Model projections of COVID-19 related mortality under different scenarios of VOC co-circulation and NPIs. (A) Example projected weekly number of deaths due to infections by each variant, assuming mRNA vaccines are used and the vaccine efficacy (VE) is as high as for the wild-type virus. Each panel shows projections for one seeding and NPI scenario, as indicated in the subtitle. Lines and surrounding areas show model projected median and interquartile range, from n = 1000 model simulations (color-coded for each variant as indicated by the legend). (B) Tallies over the entire simulation period (May – Aug 2021) for different scenarios of seeding (as indicated in the subtitles, see detail in Supplementary Table 5) and NPIs (as indicated by the x-axis labels, see detail in Supplementary Table 5). Due to the uncertainty of the infection fatality risk among breakthrough infections (i.e., those who have been vaccinated), all simulations shown here assume no reduction in VE. All numbers are scaled to per 1 million people.

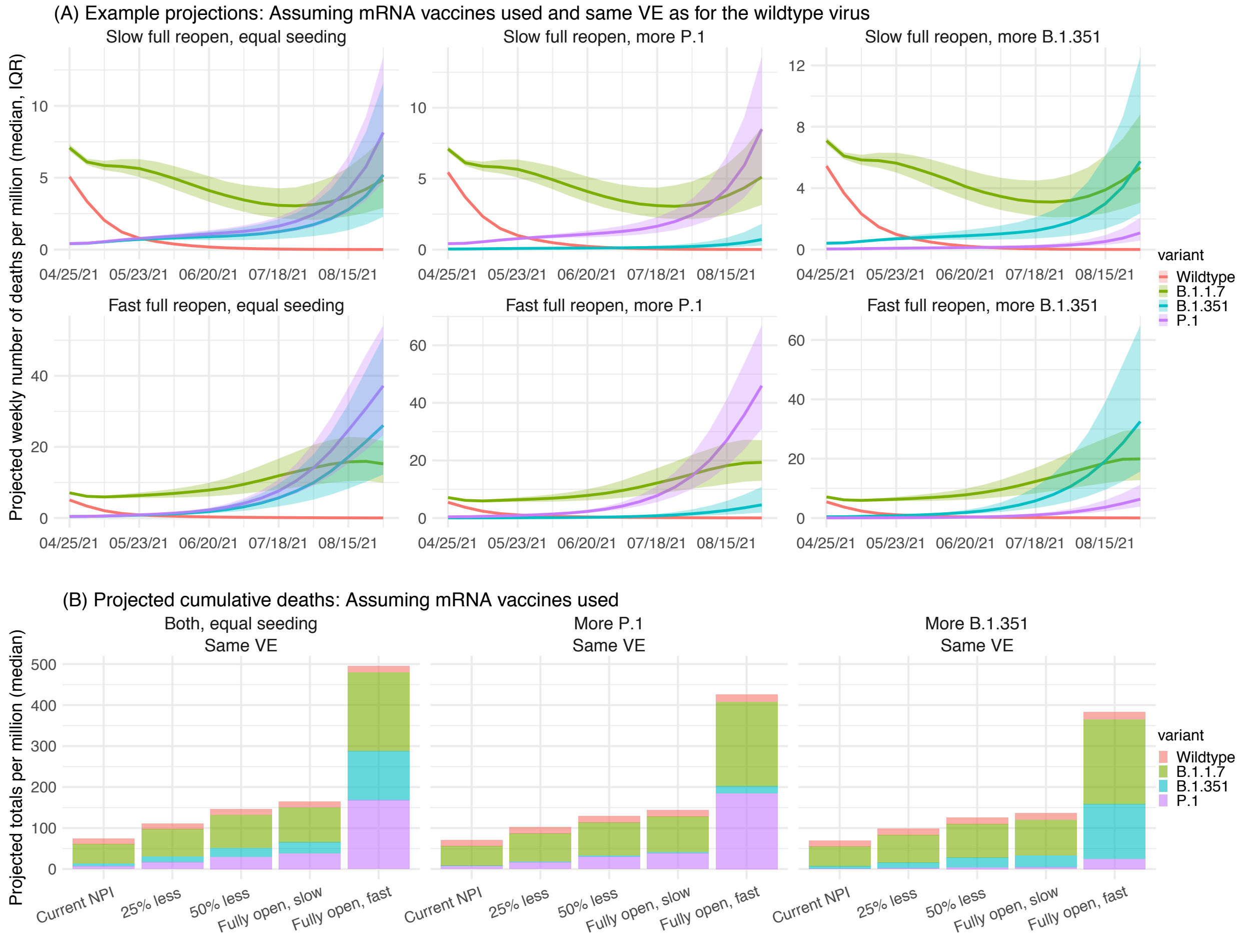

Supplementary Fig 5. Model projections of infection, under different scenarios of VOC co-circulation, NPIs, and vaccines/VEs. (A) Example projected epidemic trajectories for each variant assuming very high VE as for the mRNA vaccines against the wildtype virus. Lines and surrounding areas show model projected median and interquartile range, from n = 1000 model simulations (color-coded for each variant as indicated by the legend). (B) Tallies over the entire simulation period (May – Aug 2021) for different scenarios of seeding, VE (as indicated in the subtitles, see detail in Supplementary Table 5), and NPIs (as indicated by the x-axis labels, see detail in Supplementary Table 5). All numbers are scaled to per 1 million people.

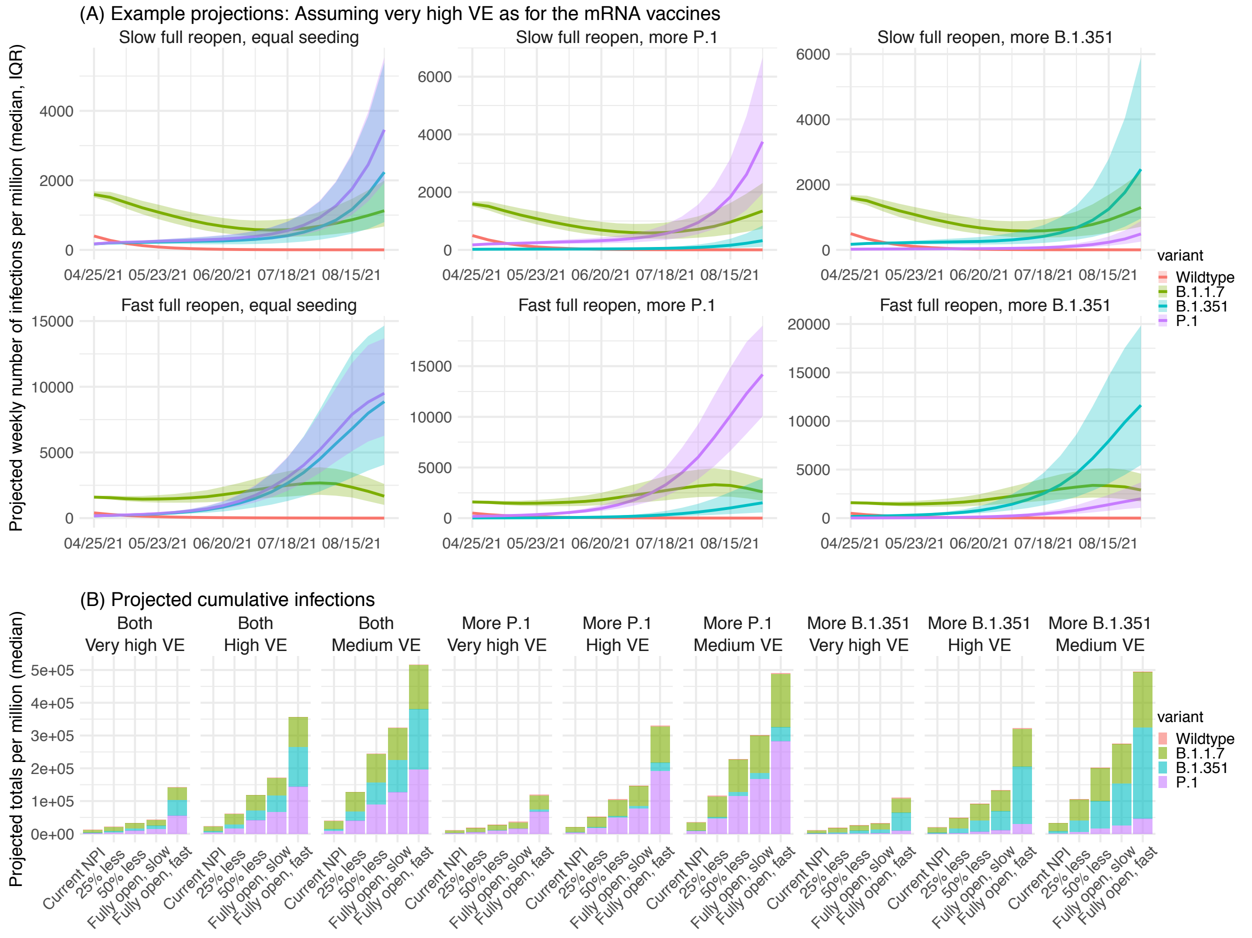

Supplementary Fig 6. Model performance for (A) United Kingdom, (B) South Africa, and (C) Brazil. Boxes (middle bar = median, box edges = interquartile range) and whiskers (95% CIs) show the distributions of relative root-mean-square-error (RRMSE) for the posterior estimates (cases in blue and deaths in pink) and one-step-ahead predictions, across n = 100 model-inference runs. See SI, Section 2.3 for details on RRMSE calculation.

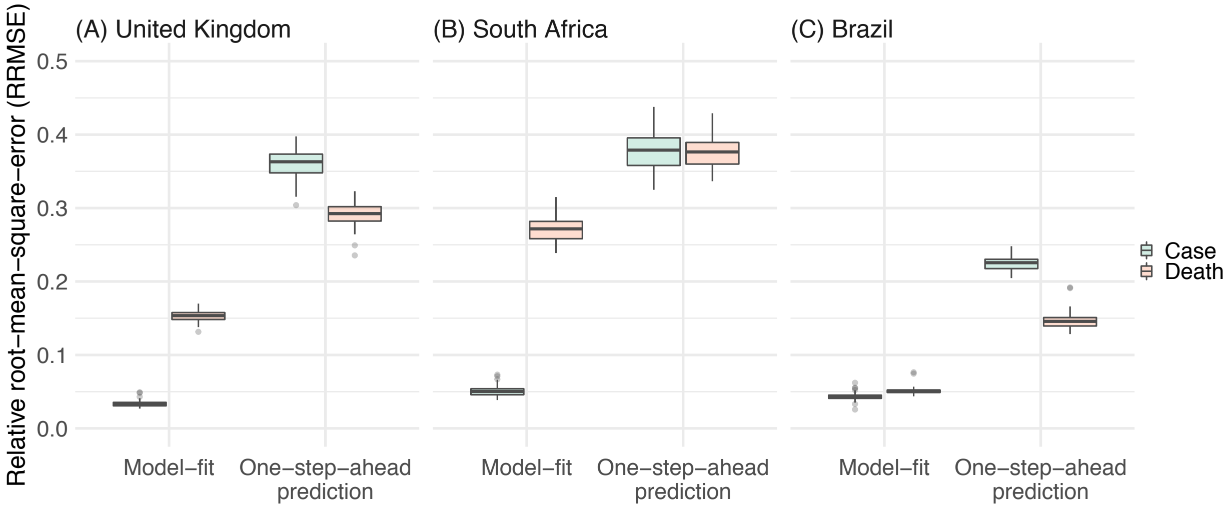

Supplementary Fig 7. Weekly average temperature and specific humidity for the three countries. These data were used to estimate the disease seasonality for each country.

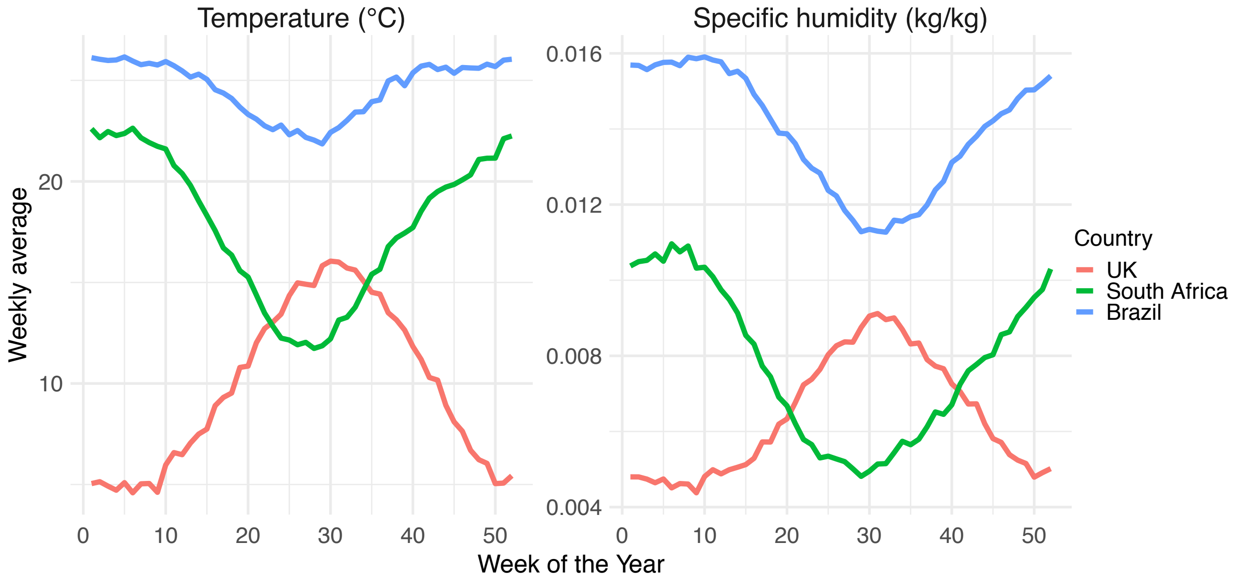

Supplementary Table 1. Model-simulated prevalence of different variants under different scenarios. Numbers show the median (and interquartile range; all in percentage) of tallies over the entire simulation period (i.e. the week of 4/25/2021 to 8/22/2021) for each scenario, as specified in columns 1 (seeding of the B.1.351 and P.1 variant), 2 (NPI), and 3 (reduction on vaccine efficacy). For all these simulations, the models assume mRNA vaccines are used and a baseline VE of 85% after the 1st dose and 95% after the 2nd dose; see details in Supplementary Table 5.

Seeding	NPI	VE	Wildtype	B.1.1.7	B.1.351	P.1
Equal seeding	Current NPI	Same VE	8.5 (7.8, 9.1)	64.3 (61.3, 67.1)	12.6 (10.1, 15.8)	13.8 (12.1, 16)
Equal seeding	Current NPI	Minor reduction	8.3 (7.6, 9)	64 (60.6, 66.9)	12.8 (10.2, 16.3)	14.1 (12.3, 16.4)
Equal seeding	Current NPI	Medium reduction	7.5 (6.9, 8.2)	63.7 (59.9, 66.7)	13.3 (10.4, 17.2)	14.5 (12.4, 17.1)
Equal seeding	Current NPI	Large reduction	7.2 (6.6, 7.9)	61 (56.8, 64.4)	15 (11.6, 19.5)	15.7 (13.5, 18.4)
Equal seeding	25% less	Same VE	5.4 (4.6, 6.2)	55.2 (49.3, 61)	16.4 (11.8, 23.7)	20.5 (16.5, 25.1)
Equal seeding	25% less	Minor reduction	5.1 (4.3, 5.9)	53.7 (47.2, 59.2)	17.7 (12.6, 25.2)	21.1 (17, 26.2)
Equal seeding	25% less	Medium reduction	4.3 (3.5, 5.1)	51.7 (44.8, 58.4)	19.9 (13, 28.9)	21.2 (16.7, 26.7)
Equal seeding	25% less	Large reduction	3.8 (3, 4.6)	45.5 (37.2, 51.6)	23.6 (15.1, 33.9)	24.9 (19.8, 31.2)
Equal seeding	50% less	Same VE	3.4 (2.7, 4.3)	44.4 (36.5, 52.3)	21.7 (13.5, 32.6)	26.4 (20.2, 34.2)
Equal seeding	50% less	Minor reduction	3.2 (2.4, 4.1)	42 (34.8, 50.1)	23.2 (14.8, 35.8)	26.7 (19.9, 34.9)
Equal seeding	50% less	Medium reduction	2.5 (1.9, 3.2)	39.9 (31.3, 47.8)	26.5 (16.5, 39.3)	27.4 (20.1, 35.9)
Equal seeding	50% less	Large reduction	2 (1.5, 2.6)	30.9 (23.6, 38.9)	31.4 (18.8, 44.1)	32 (24.8, 40.8)
Equal seeding	Fully open, slow	Same VE	2.6 (1.9, 3.4)	38 (30, 46.6)	23.2 (14.2, 36.7)	31.6 (22.4, 40.2)
Equal seeding	Fully open, slow	Minor reduction	2.3 (1.7, 3.1)	35.1 (26.7, 44.3)	26 (15.1, 41.9)	31.3 (21.8, 40.6)
Equal seeding	Fully open, slow	Medium reduction	1.8 (1.4, 2.4)	33.5 (25.2, 42)	28.4 (17, 43.8)	30.8 (22.3, 41)
Equal seeding	Fully open, slow	Large reduction	1.4 (1, 1.9)	24.4 (17.4, 31.4)	33.3 (20, 51.7)	35.9 (25, 47.9)
Equal seeding	Fully open, fast	Same VE	1 (0.8, 1.2)	25.8 (19.6, 33.8)	30.1 (17.5, 45.5)	38.1 (27.4, 50.1)
Equal seeding	Fully open, fast	Minor reduction	0.9 (0.7, 1.1)	23.4 (17.3, 30.3)	34.1 (19.7, 50.3)	38.3 (26.1, 49.8)
Equal seeding	Fully open, fast	Medium reduction	0.7 (0.6, 0.9)	22.1 (16, 30.1)	38.7 (22.6, 54)	34.5 (24.8, 47.8)
Equal seeding	Fully open, fast	Large reduction	0.6 (0.5, 0.7)	15.9 (12.1, 20.8)	41.2 (25.2, 58.1)	39.5 (27.3, 52.6)
More P.1	Current NPI	Same VE	11.7 (10.9, 12.6)	70.8 (68.6, 72.7)	1.7 (1.3, 2.3)	15.5 (13.6, 17.8)
More P.1	Current NPI	Minor reduction	11.5 (10.7, 12.3)	70.4 (68.2, 72.6)	1.8 (1.4, 2.5)	15.8 (13.7, 18.3)

More P.1	Current NPI	Medium reduction	10.5 (9.7, 11.3)	70.8 (68.3, 73.2)	1.9 (1.4, 2.6)	16.2 (14.1, 19)
More P.1	Current NPI	Large reduction	10.2 (9.4, 11.1)	69.2 (66, 71.7)	2.1 (1.5, 2.9)	18 (15.6, 21.4)
More P.1	25% less	Same VE	7.8 (6.8, 8.9)	65.1 (59.8, 68.8)	2.5 (1.6, 4)	23.9 (19.6, 29.2)
More P.1	25% less	Minor reduction	7.5 (6.6, 8.6)	63.8 (59.1, 68)	2.8 (1.7, 4.3)	25 (20.5, 30.4)
More P.1	25% less	Medium reduction	6.5 (5.6, 7.5)	63.2 (57.9, 68.4)	2.9 (1.8, 5)	26.1 (21, 32)
More P.1	25% less	Large reduction	5.8 (4.9, 6.9)	56.8 (50.5, 62.4)	3.7 (2.3, 6.5)	31.8 (26.4, 39)
More P.1	50% less	Same VE	5.3 (4.1, 6.5)	55.6 (47.9, 62.8)	3.2 (1.7, 5.6)	33.9 (26.1, 43)
More P.1	50% less	Minor reduction	4.9 (3.9, 6.2)	53.3 (46.3, 60.9)	3.5 (1.9, 6.7)	35.8 (28.7, 43.6)
More P.1	50% less	Medium reduction	4.1 (3.1, 5.2)	51.9 (43.9, 59.1)	4.2 (2.1, 8.5)	37.4 (29.5, 45.3)
More P.1	50% less	Large reduction	3.3 (2.5, 4.2)	42.3 (34.5, 50.8)	5.2 (2.6, 10.8)	46.1 (36.7, 55.1)
More P.1	Fully open, slow	Same VE	4 (3.2, 5.1)	49.8 (40.6, 57.5)	3.8 (1.9, 7.4)	40 (32.3, 50.1)
More P.1	Fully open, slow	Minor reduction	3.8 (2.9, 4.9)	46.9 (39, 55.3)	4.3 (2.2, 8.3)	42.2 (33.3, 51.1)
More P.1	Fully open, slow	Medium reduction	3 (2.3, 4)	45.6 (36.5, 53.2)	4.7 (2.4, 9.9)	44.1 (34.9, 53.5)
More P.1	Fully open, slow	Large reduction	2.3 (1.8, 3)	34.1 (26.3, 43.2)	6.2 (2.9, 12.4)	53.3 (43.2, 63.1)
More P.1	Fully open, fast	Same VE	1.4 (1.2, 1.8)	34.5 (26.3, 43.6)	5.9 (2.7, 12.3)	54.4 (44.8, 64.2)
More P.1	Fully open, fast	Minor reduction	1.3 (1.1, 1.6)	31.8 (24.6, 40.2)	6.9 (3.1, 14.5)	55.8 (46.2, 65.5)
More P.1	Fully open, fast	Medium reduction	1.1 (0.9, 1.3)	31.8 (24.9, 39.6)	7.8 (3.6, 16.2)	54.7 (45.2, 64.3)
More P.1	Fully open, fast	Large reduction	0.9 (0.7, 1)	22.7 (17.5, 29.1)	9.5 (4.4, 19.1)	63.3 (53.5, 71.3)
More B.1.351	Current NPI	Same VE	11.7 (11, 12.6)	71.9 (68.8, 74.6)	14.1 (11.3, 17.5)	1.9 (1.5, 2.3)
More B.1.351	Current NPI	Minor reduction	11.6 (10.7, 12.4)	71.5 (68, 74.2)	14.4 (11.7, 18.5)	1.9 (1.6, 2.4)
More B.1.351	Current NPI	Medium reduction	10.6 (9.7, 11.5)	71.4 (67.8, 74.8)	15.3 (12, 20)	2 (1.6, 2.5)
More B.1.351	Current NPI	Large reduction	10.2 (9.3, 11.1)	69.9 (65.4, 73.2)	17.4 (13.3, 22.4)	2.2 (1.8, 2.8)
More B.1.351	25% less	Same VE	8 (6.8, 9.2)	67.6 (60.3, 72.8)	20.2 (14.7, 29.1)	3 (2.1, 4)
More B.1.351	25% less	Minor reduction	7.6 (6.4, 8.8)	65.9 (57.6, 71.3)	22.5 (16.2, 32.4)	3 (2.2, 4.4)
More B.1.351	25% less	Medium reduction	6.6 (5.4, 7.7)	64.2 (55.6, 71.2)	25.1 (17, 35.3)	3.1 (2.2, 4.3)
More B.1.351	25% less	Large reduction	6 (4.8, 7.2)	59.1 (47.9, 66.9)	30.1 (20.7, 42.8)	3.9 (2.7, 5.6)
More B.1.351	50% less	Same VE	5.5 (4.3, 6.9)	60 (48.4, 69.1)	28.4 (18.1, 42)	4.2 (2.8, 6.5)
More B.1.351	50% less	Minor reduction	5.1 (3.9, 6.4)	57.3 (44.5, 66.5)	31.8 (21.2, 46.7)	4.2 (2.7, 6.5)
More B.1.351	50% less	Medium reduction	4.1 (3.1, 5.4)	55.2 (41.9, 65)	34.9 (22.8, 50.7)	4.5 (3, 6.9)

More B.1.351	50% less	Large reduction	3.5 (2.5, 4.6)	43.8 (32, 56.1)	45.6 (30.2, 60.6)	5.8 (3.4, 9)
More B.1.351	Fully open, slow	Same VE	4.3 (3.2, 5.8)	54.6 (42.2, 65.2)	33.7 (20.7, 48.9)	5.5 (3.4, 8.3)
More B.1.351	Fully open, slow	Minor reduction	3.9 (2.9, 5.4)	50.6 (37.6, 62.4)	38.5 (23.8, 53.3)	5.5 (3.3, 8.5)
More B.1.351	Fully open, slow	Medium reduction	3 (2.1, 4.1)	47.4 (32, 60.2)	41.3 (25.7, 60.9)	5.4 (3.1, 8.9)
More B.1.351	Fully open, slow	Large reduction	2.4 (1.6, 3.4)	36.2 (23.2, 49.6)	52.2 (34.5, 68.6)	7.4 (4.1, 12.1)
More B.1.351	Fully open, fast	Same VE	1.5 (1.2, 2)	39.9 (27.5, 52.9)	47.3 (30.6, 63.9)	8.1 (4.7, 14)
More B.1.351	Fully open, fast	Minor reduction	1.4 (1.1, 1.8)	34.3 (22.5, 47.8)	54.7 (36.4, 69.2)	7.6 (4.4, 12.7)
More B.1.351	Fully open, fast	Medium reduction	1.1 (0.9, 1.5)	33.1 (22.6, 46.6)	55.5 (38.1, 70.5)	7.8 (4.5, 13.5)
More B.1.351	Fully open, fast	Large reduction	0.9 (0.7, 1.1)	23.7 (15.7, 34.5)	64.9 (46.7, 77)	9.1 (5, 15.9)

Supplementary Table 2. Prior ranges for the parameters used in the model-inference system for the three countries.

Parameter/ variable	Symbol	Prior range	Source/rationale
Initial exposed	E(t=0)	5 – 50 times (for the UK), 1 – 10 times (for South Africa), or 2.5 – 25 times (for Brazil) of the reported number of cases on the first week of simulation	Low infection-detection rate in first weeks; likely higher initial introduction for the UK due to more international travel per capita.
Initial infectious	I(t=0)	Same as for E(t=0)	
Initial susceptible	S(t=0)	99 – 100% of the population	Almost everyone is susceptible initially
Population size	N	N/A	Based on data
Variant-specific transmission rate	β	[0.5, 0.8] for the UK; [0.4, 0.7] for South Africa; [0.4, 0.8] for Brazil	Based on R_0 estimates of around 1.5-4 for SARS-CoV-2.[20, 21, 22] Slightly lower ranges are used for South Africa and Brazil, as initial testing showed that the priors tend to overestimate the observations.
Scaling of effectiveness of NPI	e	[0.5, 1.5]	Around 1, with a large bound to be flexible.

Latency period	Z	[2, 5] days	Incubation period: 5.2 days (95% CI: 4.1, 7)[20]; latency period is likely shorter than the incubation period
Infectious period	D	[2, 5] days	Time from symptom onset to hospitalization: 3.8 days (95% CI: 0, 12.0) in China,[23] plus 1-2 days viral shedding before symptom onset. We did not distinguish symptomatic/asymptomatic infections.
Immunity period	L	[730, 1095] days	Assuming immunity lasts for 2-3 years
Mean of time from viral shedding to diagnosis	T_m	[5, 7] days for the UK, [5, 8] days for South Africa and Brazil	From a few days to a week from symptom onset to diagnosis/reporting,[23] plus 1-2 days of viral shedding (being infectious) before symptom onset. There may be a slightly longer delay for South Africa and Brazil.
Standard deviation (SD) of time from viral shedding to diagnosis	T_{sd}	[1, 3] days	To allow variation in time to diagnosis/reporting

Infection-detection rate	*r*	For the UK: starting from U[0.01, 0.15] at time 0 and allowed to increase over time using space re-probing[16] with values drawn from U[0.1, 0.24] starting at week 5 (the week of 3/29/2020), U[0.05, 0.2] during the summer (July – Aug 2020), U[0.1, 0.3] starting Sep 2020, and U[0.15, 0.3] starting Nov 2020.<br>For South Africa: starting from U[0.01, 0.06] at time 0 and allowed to change over time using space re-probing[16] with values drawn from U[0.02, 0.08] starting at week 5 (the week of 4/12/2020).<br>For Brazil: starting from U[0.01, 0.1] at time 0 and allowed to change over time using space re-probing[16] with values drawn from U[0.02, 0.1] starting at week 5 (the week of 4/12/2020).	Large uncertainties; therefore, in general we use large prior bounds and large bounds for space re-probing (SR). Note that SR is only applied to 3-10% of the ensemble members and *r* can migrate outside either the initial range or the SR ranges during EAKF update. Efforts were made in the UK to increase detection of infection; however, detection during the summer of 2020 was likely lower because more infections at the time occurred among younger age groups with no or mild symptoms. In South Africa, due to the younger age structure in the population, infection detection rates were likely lower. In Brazil, infection-detection rates were likely low throughout the pandemic.
Infection fatality risk (IFR)		For the UK: starting from U[0.001, 0.015] at time 0 and allowed to change over time using space re-probing[16] with values drawn from U[0.0001, 0.003] during Jun – Sep 2020 when infections occurred mostly among younger ages, values drawn from U[0.0001, 0.005] during Oct – Dec 2020, and [0.0001, 0.006] during Jan – Apr 2021 to account for higher IFR for B.1.1.7.<br>For South Africa: starting from [0.0001, 0.003] at time 0 and allowed to change over time using space re-probing[16]	Based on previous estimates[24] but extend to have wider ranges. Note that SR is only applied to 3-10% of the ensemble members and IFR can migrate outside either the initial range or the SR ranges during EAKF update.

	with values drawn from U[0.0001, 0.0015] during the week of 4/19/2020 to the week of 7/5/2020 when case fatality risk was lower as computed from the data, values drawn from U[0.0001, 0.002] starting the week of 12/13/2020 with the rise of B.1.351.<br>For Brazil: starting from U[0.001, 0.01] at time 0 and allowed to change over time using space re-probing[16] with values drawn from U[0.0001, 0.007] starting at week 5 (the week of 4/12/2020), values drawn from U[0.0001, 0.0035] during the week of 6/21/2020 to the week of 11/8/2020 when case fatality risk was lower as computed from the data, values drawn from U[0.0001, 0.006] starting the week of 1/3/2021 with the rise of P.1, and values drawn from U[0.0001, 0.01] starting the week of 2/7/2021 when the healthcare systems began to be overwhelmed.	
Vaccine efficacy (VE)	For the UK: VE = 85% fourteen days after the 1st dose, and 95% seven days after the 2nd dose.<br>For South Africa: VE = 60% fourteen days after the 1st dose; no 2nd dose (for J&J vaccine).<br>For Brazil: VE = 45% fourteen days after the 1st dose, and 55% seven days after the 2nd dose.	During our study period (up to mid-April 2021), the UK mostly used the Pfizer and later on Oxford/AstraZeneca vaccine, both shown to be highly effective against both the wildtype virus and B.1.1.7. South Africa mostly used the J&J vaccine with just one dose. Brazil mostly used the Sinovac and Oxford/AstraZeneca vaccines with relatively lower VE against P.1.

Supplementary Table 3. Parameters used to generate the synthetic data for model validation

Truth	Settings
Wildtype-virus, same for all truths	β = 0.65 per day; Z = 3.5 days; D = 3.5 days; L = 2.5 years; T_m = 6 days; T_{sd} = 2 days; IFR = 0.7%
Variant in Truth 1	Large outbreak during the first wave (See Fig 1 and Supplementary Fig 1). No increase in transmissibility (i.e. same β and D as for the wildtype virus); 80% increase in immune escape. Other parameters same as the wildtype virus.
Variant in Truth 2	Large outbreak during the first wave (See Fig 1 and Supplementary Fig 1). 50% increase in transmissibility (i.e. β = 0.65 x 1.5 = 0.975 per day); no immune escape. Other parameters same as the wildtype virus.
Variant in Truth 3	Large outbreak during the first wave (See Fig 1 and Supplementary Fig 1). 50% increase in transmissibility (i.e. β = 0.65 x 1.5 = 0.975 per day); 80% increase in immune escape. Other parameters same as the wildtype virus.
Variant in Truth 4	Large outbreak during the first wave (See Fig 1 and Supplementary Fig 1). 25% increase in transmissibility (i.e. β = 0.65 x 1.25 = 0.8125 per day); 40% increase in immune escape. Other parameters same as the wildtype virus.
Variant in Truth 5	Small outbreak during the first wave (See Fig 1 and Supplementary Fig 1). 50% increase in transmissibility (i.e. β = 0.65 x 1.5 = 0.975 per day); no immune escape. Other parameters same as the wildtype virus.
Infection-detection rate	20% for results shown in Fig 1 and 10% for results shown in Supplementary Fig 1.

Supplementary Table 4. Cumulative vaccination coverage used in the multi-variant, age-structured model simulations. Baseline vaccination coverage (as of 4/24/2021) is based on data for NYC. Projected vaccination rates for the simulation period (from the week starting 4/25/2021 to the week ending 8/29/2021) are based on data 10 days preceding the simulation and assuming a cumulative vaccination uptake of 80%. Note that ages under 15 (i.e., <1, 1-4, 5-14 years) are combined in the same column as they were not eligible to receive the vaccines at the time of this study. In addition, the numbers are aggregated from neighborhood-level estimates and thus could slightly exceed the 80% total for some age groups when some neighborhoods had actual vaccination coverage above 80% at baseline.

Time point	Vaccine	Vaccination coverage (%) by age group (in year)					
	dose	0-14	15-24	25-44	45-64	65-74	75+
Start of simulation:	1st dose	0	25.4	41.7	52.9	69	58.2
4/24/2021	2nd dose	0	10.5	26.7	40.7	62.6	53.6
End of simulation:	1st dose	0	70	76	79.1	80.6	76.8
8/29/2021	2nd dose	0	65.4	73.4	78.7	80.3	74.5

Supplementary Table 5. Parameter settings for different scenarios to simulate and project the impact of different variants of concern. Initial conditions and parameters are randomly drawn from uniform distributions with lower and upper bounds as specified below, based on estimates for NYC made for the week of 4/18/2021 using data during 3/1/2020 – 4/24/2021. Numbers associated with the parameter names denote the corresponding age groups.

scenario: Seeding/VE/NPI	variant	parameter	lower bound	upper bound
Equal seeding	wildtype	Initial seeding (%)	45	61
Equal seeding	B.1.1.7	Initial seeding (%)	35	45
Equal seeding	B.1.351	Initial seeding (%)	2	5
Equal seeding	P.1	Initial seeding (%)	2	5
More B.1.351	wildtype	Initial seeding (%)	49.4	62.8
More B.1.351	B.1.1.7	Initial seeding (%)	35	45
More B.1.351	B.1.351	Initial seeding (%)	2	5
More B.1.351	P.1	Initial seeding (%)	0.2	0.6
More P.1	wildtype	Initial seeding (%)	49.4	62.8
More P.1	B.1.1.7	Initial seeding (%)	35	45
More P.1	B.1.351	Initial seeding (%)	0.2	0.6
More P.1	P.1	Initial seeding (%)	2	5
All	All	Travel-related importation, ε_i	1 per 21 days, for the entire city (N = 8.4 million)	
Same VE (as mRNA vaccines against wildtype) or Very high VE	wildtype	VE = 85% fourteen days after the 1st dose and 95% seven days after the 2nd days		
	B.1.1.7	VE = 85% fourteen days after the 1st dose and 95% seven days after the 2nd dose		
	B.1.351	VE = 85% fourteen days after the 1st dose and 95% seven days after the 2nd dose		
	P.1	VE = 85% fourteen days after the 1st dose and 95% seven days after the 2nd dose		
Minor reduction in VE (mRNA vaccines)	wildtype	VE = 85% fourteen days after the 1st dose and 95% seven days after the 2nd dose		
	B.1.1.7	VE = 85% x .95 = 80.75% fourteen days after the 1st dose and 95% seven days after the 2nd dose		
	B.1.351	VE = 85% x .80 = 68% fourteen days after the 1st dose and VE = 95% x .9 = 85.5% seven days after the 2nd dose		

	P.1	VE = 85% x .85 = 72.25% fourteen days after the 1st dose and VE = 95% x .95 = 90.25% seven days after the 2nd dose		
Medium reduction in VE (mRNA vaccines)	wildtype	VE = 85% fourteen days after the 1st dose and 95% seven days after the 2nd dose		
	B.1.1.7	VE = 85% x .95 = 80.75% fourteen days after the 1st dose and VE = 95% x .95 = 90.25% seven days after the 2nd dose		
	B.1.351	VE = 85% x .70 = 59.5% fourteen days after the 1st dose and VE = 95% x .8 = 76% seven days after the 2nd dose		
	P.1	VE = 85% x .8 = 68% fourteen days after the 1st dose and VE = 95% x .9 = 85.5% seven days after the 2nd dose		
Large reduction in VE (mRNA vaccines)	wildtype	VE = 85% fourteen days after the 1st dose and 95% seven days after the 2nd dose		
	B.1.1.7	VE = 85% x .95 = 80.75% fourteen days after the 1st dose and VE = 95% x .95 = 90.25% seven days after the 2nd dose		
	B.1.351	VE = 85% x .5 = 42.5% fourteen days after the 1st dose and VE = 95% x .6 = 57% seven days after the 2nd dose		
	P.1	VE = 85% x .6 = 51% fourteen days after the 1st dose and VE = 95% x .7 = 66.5% seven days after the 2nd dose		
High VE	All variants	VE = 60% fourteen days after the 1st dose and VE = 70% seven days after the 2nd dose		
Medium VE	All variants	VE = 45% fourteen days after the 1st dose and VE = 55% seven days after the 2nd dose		
Current NPI	all variants	no further increase in transmission rate		
25% less NPI	all variants	transmission rate increases by 5% per week up to 25% in total		
50% less NPI	all variants	transmission rate increases by 5% per week up to 50% in total		
Full reopen, slow	all variants	transmission rate increases by 5% per week up to fully reopen (~85% of current level)		
Full reopen, fast	all variants	transmission rate increases by 10% per week up to fully reopen (~85% of current level)		
	wildtype	β_{11} (per day, same below)	0.11	0.16
	wildtype	β_{22}	0.078	0.11
	wildtype	β_{33}	0.11	0.15
	wildtype	β_{44}	0.13	0.19
	wildtype	β_{55}	0.16	0.25
	wildtype	β_{66}	0.13	0.19

wildtype	β_{77}	0.13	0.19
wildtype	β_{88}	0.11	0.16
wildtype	β_{12}	0.055	0.08
wildtype	β_{13}	0.014	0.021
wildtype	β_{14}	0.0057	0.0084
wildtype	β_{15}	0.018	0.026
wildtype	β_{16}	0.0082	0.012
wildtype	β_{17}	0.0058	0.0085
wildtype	β_{18}	0.004	0.0059
wildtype	β_{21}	0.039	0.055
wildtype	β_{23}	0.01	0.014
wildtype	β_{24}	0.0041	0.0058
wildtype	β_{25}	0.013	0.018
wildtype	β_{26}	0.0059	0.0083
wildtype	β_{27}	0.0042	0.0059
wildtype	β_{28}	0.0029	0.004
wildtype	β_{31}	0.013	0.018
wildtype	β_{32}	0.013	0.018
wildtype	β_{34}	0.0066	0.0089
wildtype	β_{35}	0.0087	0.012
wildtype	β_{36}	0.0048	0.0066
wildtype	β_{37}	0.0034	0.0046
wildtype	β_{38}	0.0044	0.006
wildtype	β_{41}	0.0073	0.011
wildtype	β_{42}	0.0073	0.011
wildtype	β_{43}	0.012	0.017
wildtype	β_{45}	0.012	0.017
wildtype	β_{46}	0.0089	0.013
wildtype	β_{47}	0.0032	0.0046
wildtype	β_{48}	0.0059	0.0086
wildtype	β_{51}	0.075	0.12
wildtype	β_{52}	0.075	0.12
wildtype	β_{53}	0.061	0.096
wildtype	β_{54}	0.048	0.076
wildtype	β_{56}	0.046	0.073
wildtype	β_{57}	0.028	0.043
wildtype	β_{58}	0.027	0.042

wildtype	β_{61}	0.04	0.058
wildtype	β_{62}	0.04	0.058
wildtype	β_{63}	0.033	0.047
wildtype	β_{64}	0.039	0.056
wildtype	β_{65}	0.04	0.058
wildtype	β_{67}	0.044	0.064
wildtype	β_{68}	0.04	0.058
wildtype	β_{71}	0.024	0.035
wildtype	β_{72}	0.024	0.035
wildtype	β_{73}	0.017	0.025
wildtype	β_{74}	0.0074	0.011
wildtype	β_{75}	0.018	0.025
wildtype	β_{76}	0.025	0.036
wildtype	β_{78}	0.051	0.074
wildtype	β_{81}	0.017	0.024
wildtype	β_{82}	0.017	0.024
wildtype	β_{83}	0.018	0.025
wildtype	β_{84}	0.013	0.018
wildtype	β_{85}	0.016	0.023
wildtype	β_{86}	0.028	0.04
wildtype	β_{87}	0.043	0.061
wildtype	Z_1 (days, same below)	3.3	4.3
wildtype	Z_2	3.3	4.3
wildtype	Z_3	3.4	4.4
wildtype	Z_4	3.4	4.4
wildtype	Z_5	3.5	4.4
wildtype	Z_6	3.5	4.5
wildtype	Z_7	3.4	4.5
wildtype	Z_8	3.3	4.4
wildtype	D_1	3.1	4
wildtype	D_2	3.1	4.1
wildtype	D_3	3	4
wildtype	D_4	3.3	4.2
wildtype	D_5	3.2	4.2
wildtype	D_6	3.3	4.2
wildtype	D_7	3.5	4.4
wildtype	D_8	3.3	4.3

	wildtype	IFR_1	7.70E-05	0.00012
	wildtype	IFR_2	7.60E-05	0.00013
	wildtype	IFR_3	7.70E-05	0.00012
	wildtype	IFR_4	7.60E-05	0.00013
	wildtype	IFR_5	0.00031	0.00049
	wildtype	IFR_6	0.0033	0.0047
	wildtype	IFR_7	0.018	0.023
	wildtype	IFR_8	0.056	0.069
	B.1.1.7	Increase in transmission rate	0.403	0.5227
	B.1.1.7	Immune escape	0	0.1
	B.1.351	Increase in transmission rate	0.1849	0.457
	B.1.351	Immune escape	0.4414	0.8281
	P.1	Increase in transmission rate	0.3682	0.4945
	P.1	Immune escape	0.3588	0.6683
	B.1.1.7	Increase in transmission rate	0.403	0.5227
	B.1.1.7	Immune escape	0	0.1
	B.1.351	Increase in transmission rate	0.1849	0.457
	B.1.351	Immune escape	0.4414	0.8281
	P.1	Increase in transmission rate	0.3682	0.4945
	P.1	Immune escape	0.3588	0.6683

Supplementary References:

1. Moriyama M, Hugentobler WJ, Iwasaki A. Seasonality of Respiratory Viral Infections. *Annu Rev Virol* **7**, 83-101 (2020).
2. Tamerius J, Nelson MI, Zhou SZ, Viboud C, Miller MA, Alonso WJ. Global influenza seasonality: reconciling patterns across temperate and tropical regions. *Environ Health Perspect* **119**, 439-445 (2011).
3. Baker RE*, et al.* Epidemic dynamics of respiratory syncytial virus in current and future climates. *Nat Commun* **10**, 5512 (2019).
4. Kissler SM, Tedijanto C, Goldstein E, Grad YH, Lipsitch M. Projecting the transmission dynamics of SARS-CoV-2 through the postpandemic period. *Science* **368**, 860-868 (2020).
5. Tamerius JD*, et al.* Environmental predictors of seasonal influenza epidemics across temperate and tropical climates. *PLoS Pathog* **9**, e1003194 (2013).
6. Shaman J, Kohn M. Absolute humidity modulates influenza survival, transmission, and seasonality. *Proc Natl Acad Sci U S A* **106**, 3243-3248 (2009).
7. Shaman J, Pitzer VE, Viboud C, Grenfell BT, Lipsitch M. Absolute humidity and the seasonal onset of influenza in the continental United States. *Plos Biol* **8**, e1000316 (2010).
8. Yang W, Marr LC. Mechanisms by which ambient humidity may affect viruses in aerosols. *Appl Environ Microbiol* **78**, 6781-6788 (2012).
9. Yang W, Shaff J, Shaman J. Effectiveness of non-pharmaceutical interventions to contain COVID-19: a case study of the 2020 spring pandemic wave in New York City. *J R Soc Interface* **18**, 20200822 (2021).
10. Yang W*, et al.* Estimating the infection-fatality risk of SARS-CoV-2 in New York City during the spring 2020 pandemic wave: a model-based analysis. *The Lancet Infectious diseases* **21**, 203-212 (2021).
11. Yuan H, Kramer SC, Lau EHY, Cowling BJ, Yang W. Modeling influenza seasonality in the tropics and subtropics. *PLoS Comput Biol* **17**, e1009050 (2021).
12. Anderson JL. An ensemble adjustment Kalman filter for data assimilation. *Mon Weather Rev* **129**, 2884-2903 (2001).
13. Lasry A*, et al.* Timing of Community Mitigation and Changes in Reported COVID-19 and Community Mobility - Four U.S. Metropolitan Areas, February 26-April 1, 2020. *MMWR Morbidity and mortality weekly report* **69**, 451-457 (2020).
14. Kraemer MUG*, et al.* The effect of human mobility and control measures on the COVID-19 epidemic in China. *Science* **368**, 493-497 (2020).
15. Google Inc. Community Mobility Reports. (2020).
16. Yang W, Shaman J. A simple modification for improving inference of non-linear dynamical systems. *arXiv*, 1403.6804 (2014).

17. Yang W, Lau EHY, Cowling BJ. Dynamic interactions of influenza viruses in Hong Kong during 1998-2018. *PLoS Comput Biol* **16**, e1007989 (2020).
18. Gog JR, Grenfell BT. Dynamics and selection of many-strain pathogens. *Proc Natl Acad Sci U S A* **99**, 17209-17214 (2002).
19. Yang W, Kandula S, Shaman J. Projections of COVID19 Epidemic Outcomes and Healthcare Demands for New York City (NYC). (2020).
20. Li Q*, et al.* Early Transmission Dynamics in Wuhan, China, of Novel Coronavirus–Infected Pneumonia. *New Engl J Med*, **382**, 1199-1207 (2020).
21. Wu JT, Leung K, Leung GM. Nowcasting and forecasting the potential domestic and international spread of the 2019-nCoV outbreak originating in Wuhan, China: a modelling study. *Lancet* **395**, 689-697 (2020).
22. Li R*, et al.* Substantial undocumented infection facilitates the rapid dissemination of novel coronavirus (SARS-CoV-2). *Science* **368**, 489-493 (2020).
23. Zhang J*, et al.* Evolving epidemiology and transmission dynamics of coronavirus disease 2019 outside Hubei province, China: a descriptive and modelling study. *The Lancet Infectious diseases* **20**, 793-802 (2020).
24. Verity R*, et al.* Estimates of the severity of coronavirus disease 2019: a model-based analysis. *The Lancet Infectious diseases* **20**, 669-677 (2020).
