# Supplementary material for: Development of a model-inference system for estimating epidemiological characteristics of SARS-CoV-2 variants of concern
Source: Nat Commun. 2021 Sep 22;12:5573. doi: 10.1038/s41467-021-25913-9 (PMC8458278; doi:10.1038/s41467-021-25913-9)
Supplement: Supplementary file 3 — Reporting Summary [file 41467_2021_25913_MOESM3_ESM.pdf]

nature research

Corresponding author(s): Wan Yang

Last updated by author(s): Aug 19, 2021

Reporting Summary

Nature Research wishes to improve the reproducibility of the work that we publish. This form provides structure for consistency and transparency in reporting. For further information on Nature Research policies, see our Editorial Policies and the Editorial Policy Checklist.

Statistics

For all statistical analyses, confirm that the following items are present in the figure legend, table legend, main text, or Methods section.

n/a	Confirmed	
☐	☒	The exact sample size (*n*) for each experimental group/condition, given as a discrete number and unit of measurement
☐	☒	A statement on whether measurements were taken from distinct samples or whether the same sample was measured repeatedly
☒	☐	The statistical test(s) used AND whether they are one- or two-sided<br>*Only common tests should be described solely by name; describe more complex techniques in the Methods section.*
☒	☐	A description of all covariates tested
☒	☐	A description of any assumptions or corrections, such as tests of normality and adjustment for multiple comparisons
☐	☒	A full description of the statistical parameters including central tendency (e.g. means) or other basic estimates (e.g. regression coefficient) AND variation (e.g. standard deviation) or associated estimates of uncertainty (e.g. confidence intervals)
☒	☐	For null hypothesis testing, the test statistic (e.g. *F*, *t*, *r*) with confidence intervals, effect sizes, degrees of freedom and *P* value noted<br>*Give P values as exact values whenever suitable.*
☐	☒	For Bayesian analysis, information on the choice of priors and Markov chain Monte Carlo settings
☒	☐	For hierarchical and complex designs, identification of the appropriate level for tests and full reporting of outcomes
☒	☐	Estimates of effect sizes (e.g. Cohen's *d*, Pearson's *r*), indicating how they were calculated

Our web collection on statistics for biologists contains articles on many of the points above.

Software and code

Policy information about availability of computer code

Data collection	Temperature and humidity data were downloaded using the “stationaRy” R package (version 0.5.1). Model code to compile all datasets used in this study is made publicly available on Github: https://github.com/wan-yang/covid_voc_study
Data analysis	Model code to reproduce the study is made publicly available on Github: https://github.com/wan-yang/covid_voc_study

For manuscripts utilizing custom algorithms or software that are central to the research but not yet described in published literature, software must be made available to editors and reviewers. We strongly encourage code deposition in a community repository (e.g. GitHub). See the Nature Research guidelines for submitting code & software for further information.

Data

Policy information about availability of data

All manuscripts must include a data availability statement. This statement should provide the following information, where applicable:

- Accession codes, unique identifiers, or web links for publicly available datasets
- A list of figures that have associated raw data
- A description of any restrictions on data availability

All data used in this study are publicly available as described in the “Data sources and processing” section. Compiled datasets used in this study are available at https://github.com/wan-yang/covid_voc_study/tree/main/data

Field-specific reporting

Please select the one below that is the best fit for your research. If you are not sure, read the appropriate sections before making your selection.

☐ Life sciences ☐ Behavioural & social sciences ☒ Ecological, evolutionary & environmental sciences

For a reference copy of the document with all sections, see nature.com/documents/nr-reporting-summary-flat.pdf

Ecological, evolutionary & environmental sciences study design

All studies must disclose on these points even when the disclosure is negative.

Study description	This study develops a model-inference system to estimate the relative change in transmissibility and level of immune evasion for different SARS-CoV-2 variants, while accounting for under-detection of infection, delays of reporting, disease seasonality, non-pharmaceutical interventions (NPIs), and vaccination. The model-inference system is then applied to study three SARS-CoV-2 variants of concern, including B.1.1.7, B.1.351, and P.1 based on data collected in the three countries they were first identified (i.e., the UK, South Africa, and Brazil, respectively).
Research sample	N/A
Sampling strategy	N/A
Data collection	The model-inference system uses reported COVID-19 case and mortality data to capture transmission dynamics, weather data to estimate disease seasonality, mobility data to represent concurrent NPIs, and vaccination data to account for changes in population susceptibility due to vaccination. All data used in this study are publicly available as described in the "Data sources and processing" section. Compiled datasets used in this study are available at https://github.com/wan-yang/covid_voc_study/tree/main/data
Timing and spatial scale	Timing: From the week of March 1, 2020 (for the UK) or March 15, 2020 (for South Africa and Brazil) to the week of April 11, 2021.<br>Spatial scale: country-level
Data exclusions	None.
Reproducibility	Model code and relevant data to reproduce the study are made publicly available on Github: https://github.com/wan-yang/covid_voc_study
Randomization	Not applicable, because the study sites were based on where the variants of concern first emerged and had large impacts.
Blinding	Not applicable, because the study sites were based on where the variants of concern first emerged and had large impacts.

Did the study involve field work? ☐ Yes ☒ No

Reporting for specific materials, systems and methods

We require information from authors about some types of materials, experimental systems and methods used in many studies. Here, indicate whether each material, system or method listed is relevant to your study. If you are not sure if a list item applies to your research, read the appropriate section before selecting a response.

Materials & experimental systems

n/a	Involved in the study	
☒	☐	Antibodies
☒	☐	Eukaryotic cell lines
☒	☐	Palaeontology and archaeology
☒	☐	Animals and other organisms
☒	☐	Human research participants
☒	☐	Clinical data
☒	☐	Dual use research of concern

Methods

n/a	Involved in the study	
☒	☐	ChIP-seq
☒	☐	Flow cytometry
☒	☐	MRI-based neuroimaging
